# Computer vision syndrome and ergonomic risk factors among workers of the Commercial Bank of Ethiopia in Addis Ababa, Ethiopia: an institutional-based cross-sectional study

**DOI:** 10.3389/fpubh.2024.1341031

**Published:** 2024-05-09

**Authors:** Kassahun Ayele Gasheya, Azanaw Asega Belay, Teferi Abegaze, Yifokire Tefera Zele, Chala Daba

**Affiliations:** ^1^Department of Occupational Health and Safety, College of Medicine and Health Science, Wollo University, Dessie, Ethiopia; ^2^Department of Preventive Medicine, School of Public Health, Addis Ababa University, Addis Ababa, Ethiopia; ^3^Department of Environmental Health, College of Medicine and Health Science, Wollo University, Dessie, Ethiopia

**Keywords:** computer vision syndrome, ergonomic risk factors, workstation ergonomic setup, bank worker, Addis Ababa

## Abstract

**Background:**

Computer vision syndrome (CVS) is the most pressing public health concern that affects vision and reduces quality of life and productivity, particularly in developing countries. Most of the previous studies conducted in Ethiopia focus on the knowledge and personal risk factors of bank workers. Moreover, ergonomic workstation design was not objectively assessed, which could hinder the implementation of effective intervention strategies. Therefore, this study aimed to determine CVS and ergonomic factors among commercial bank workers in Addis Ababa, Ethiopia.

**Methods:**

An institutional-based cross-sectional study was carried out among 466 study participants from May 26 to July 24, 2022. A multistage sampling technique was applied to select the study participants. Data were collected via a standardized tool of CVS (CVS-Q). Besides, workstation ergonomics were pertinently assessed. The collected data was entered into EpiData version 3.1 and exported to SPSS version 26 for data analysis and cleaning. Multivariable logistics regression analysis was performed to identify factors associated with CVS. The variables with a *p*-value < 0.05 were considered statistically significant factors.

**Results:**

Prevalence of CVS was 75.3% (95% CI: 71.2–79.2%). Blurred vision, eye redness, and headache, 59.8%, 53.7%, and 50.7%, respectively, were frequently reported symptoms. Glare (AOR = 4.45: 95% CI: 2.45–8.08), 20–20–20 principle (AOR = 1.98, 95% CI: 1.06–3.67), wearing non-prescription eyeglasses (AOR = 4.17; 95% CI: 1.92–9.06), and poor workstation (AOR = 7.39; 95% CI: 4.05–13.49) was significantly associated with CVS.

**Conclusion:**

The prevalence of CVS was found to be high. Glare at work, ignoring the 20–20–20 principle, wearing non-prescription eyeglasses, and poor workstation ergonomic design were independent predictors of CVS. Therefore, comprehensive interventional activities like adhering to the 20–20–20 principle, avoiding the use of non-prescription glasses, minimizing glare, and improving workstation ergonomic setup are essential to prevent CVS.

## Introduction

The use of computer devices in the workplace, such as in banking systems, is an integral part of daily life (1). However, it has been associated with health-related problems collectively known as computer vision syndrome (CVS) (2). CVS is defined by the American Optometric Association as a collection of eye discomfort and vision problems such as eye strain (fatigue), blurred vision, excessive tearing, double vision, headache, light sensitivity, dry eye, and irritated eye that occurs when using computers for extended periods (3).

CVS is one of the most pressing public health concerns in the present time period, which diminishes visual abilities, enhances error rates, lowers workplace productivity, reduces the quality of life, and reduces job satisfaction (4–6). From the global report, data prevalence of computer users (7–9) of CVS ranges from 64 to 90%. A total of 70 million workers worldwide are at risk of developing CVS, with one million new cases occurring per year (10, 11). CVS is more prevalent in developing countries than in developed countries because of a lack of availability and use of personal protective equipment, a large workload, and insufficient break time when using a computer (12). Different scholars reported that the prevalence of CVS was 89.9% in Malaysia, 81.9% in India, 89.4% in Nepal, and 67.4% in Sri Lanka (4, 13–15), and 90% of university students experience visual pain while working on computer-aided design with high computer brightness in Italy (16). In Africa, the prevalence of CVS was found to be high. For instance, evidence from different studies showed that the magnitude of CVS was as high as 85.2% in Egypt, 74% in Abuja, Nigeria, and 51.1% in Ghana (17–19).

CVS is a significant cause of morbidity among different workers in Ethiopia. The prevalence of CVS ranged from 68.8% (20) to 81.3% (21) across different study groups. According to the subgroup analysis by occupation, bank workers had the highest CVS prevalence (5). Evidence from different studies in Ethiopia showed that the magnitude of CVS was found to be as high as 73% in Gondar City (22), 74.6% in Addis Ababa (11, 22), 76.6% in Jimma University (23), 73.9% among Gondar University workers (24), 68.8% in Addis Ababa (20), 69.5% in Debre Tabor Town (1), 70.4% among university instructors (25), and 81.3% among workers of the Ethiopian Roads Authority (21). The interaction of users with the computer, computer work, computer room conditions, computer screens, and the human eye contribute to the development of CVS caused by extended computer usage (26). Concerning office workstation ergonomics assessments, 79.5%−88.4% of computer users were working under poor workstation ergonomics (4, 18).

Evidence from aforementioned studies showed that age, gender, marital status, monthly income, educational status, and work experience in computer usage (1, 24, 27, 28), history of eye illness, frequent eye blinking, wearing eyeglasses, use of antiglare for computer screens, utilization of lubricant eye drops, taking frequent healthy breaks, duration of computer usage per day, and adjusting the brightness of computer screen (1, 3, 4, 8, 13, 14, 20, 22, 28–33), presence of glare or bright light, following 20–20–20 ergonomic principle, poor workstation ergonomics setup, ergonomically adjustable sitting chair, and keyboard (4, 6, 18, 21, 34, 35) were factors associated with CVS.

The majority of previous studies classified CVS as the presence of at least one of the abovementioned symptom (1, 11, 22, 23). Furthermore, most of these previous studies were focused on the knowledge of bank workers about CVS and personal risk factors (5, 11, 18, 22, 36), which could hinder the implementation of effective intervention strategies. However, the prevalence of CVS and ergonomics risk factors has not yet been investigated, and workstation ergonomics have not been objectively assessed in the previous investigations. Therefore, this study was conducted to determine the prevalence of CVS and identify ergonomics risk factors among workers of the Commercial Bank of Ethiopia (CBE) in Addis Ababa. The findings of this study could close a research gap by considering ergonomic risk factors and conducting workstation ergonomic assessments. It will also provide input for academicians, bank managers, optometrist nurses, and occupational health specialists to prevent and control CVS among bank workers.

## Methods and materials

### Study area

This study was conducted in the Commercial Bank of Ethiopia (CBE), Addis Ababa—the capital city of Ethiopia. CBE is the largest bank in Ethiopia, with the most number of employees and over 1,900 branches across the country, and contributes significantly to the country's economic success and development.

### Study design and period

Institutional-based cross-sectional study design was carried out to determine CVS and ergonomic factors among commercial bank workers from 26 May 2022 to 24 July 2022.

### Source and study population

All workers of CBE from four districts in Addis Ababa city were the source population of the study. All randomly selected workers of the CBE in the western and northern districts in Addis Ababa city were the study population.

### Inclusion and exclusion criteria

All workers of CBE who are using computer devices for at least 2 h per day every week and had utilized a computer for at least 1 year were eligible for inclusion in this study. However, the bank workers who were on sick leave, annual leave, maternity leave, and had preexisting eye problems like acute, chronic conjunctivitis, eyelid disorder, cataract, glaucoma, and uncorrected refractive error caused by other medical problems were excluded from the study (22, 23).

### Sample size determination

The sample size was computed by using a single population proportion formula with the assumptions: *n* = sample size, *P* (referring to the prevalence of CVS) = 74.6%, which was taken from a study conducted in Addis Ababa (11), 95% confidence interval, 5% margin of error (*d*), and critical value (*Z*_α/2_ = 1.96).


$$\begin{array}{l} n\;={\left(Z_{\alpha /2}\right)}^{2}p(1\;-\;p)/d^{2}\\ \;={\left(1.96\right)}^{2}\times 0.75(1\;-\;0.75)/{\left(0.05\right)}^{2}=291. \end{array}$$

The sample size becomes 466 after considering the design effect of 1.5 and 10% of non-response rate. The final sample size was 466.

### Sampling technique and procedures

**Figure 1:**
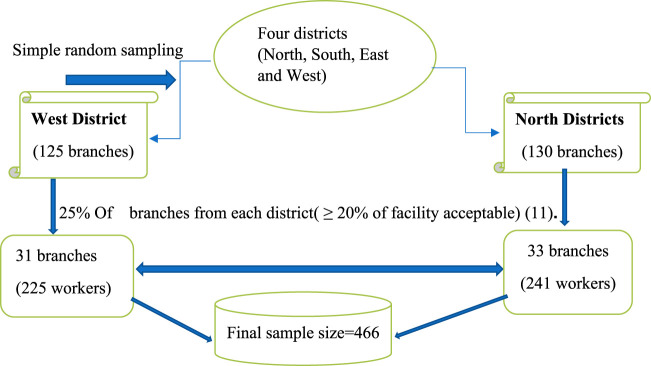
Schematic representation of the sampling procedure for the study of CVS among workers of Commercial Bank of Ethiopia, Addis Ababa, Ethiopia.

Multistage sampling techniques were applied to select study participants. A three-stage sampling technique was employed; in the first stage, two districts (North and West districts with 255 branches) were selected using simple random sampling. In the second stage with the assumption of 25% of the branches, a total of 64 branches (31 and 33 branches from western and northern districts respectively) were selected using simple random sampling methods. Following branch selection, the study participants' samples were distributed proportionally to the number of branches. In the third stage, workers from each CBE branch were selected using simple random sampling (Figure 1).

### Data collection method

#### Data collection via self-administered questionnaires

The data were collected using pretested self-administered structured questionnaires comprised of sociodemographic characteristics, personal and behavioral factors, and ergonomic and environmental factors. The prevalence of CVS was measured using a standardized CVS-Q tool (37) with 16 items (Cronbach alpha coefficients = 0.87), which assesses the internal consistency and reliability of a group of survey items to check whether a group of items consistently measures the same feature with a level of agreement on a standardized 0–1 scale. Therefore, these indicate that there was greater agreement between items from the Rasch Analysis, with sensitivity and specificity values >70%. In addition, the questionnaire has a strong test–retest repeatability, as measured by the intraclass correlation coefficient (ICC = 0.802; 95% CI, 0.673 – 0.884) and Cohen's kappa (*k* = 0.612; 95% CI, 0.384 – 0.839), demonstrating that the questionnaire is effective at detecting CVS. For all of these reasons, it is a valid and reliable instrument for assessing self-reported CVS among computer device users (38). It has frequency and severity categories (37). Participants were asked to rate how frequently they felt for each of the 16 symptoms that occurred using the following options: Never = 0, Occasionally or Sometimes = 1, and Always or Often = 2. Similarly, participants were asked to grade the severity of the perceived symptom using either Moderate = 1 or Severe = 2. Symptoms that were reported as never occurring were assigned a score of 0 (None) on the intensity scale.

#### Data collection via workstation ergonomics assessments by instrument

The second step involves observing and measuring workstation ergonomic parameters at each bank worker's workstation. A calibrated digital light meter (Standard ST-1300) was used to measure illumination around the computer workstation in three positions: left, center, and right of the working area, with a distance between extreme points not exceeding 117 cm (maximum width of the working area) (38). Then, the average of three measurements was taken at each workstation. Ten parameters, including horizontal viewing distance to the top of the screen (18–28 cm), horizontal viewing distance to the bottom of the screen (40–60 cm), horizontal viewing distance to the center of the screen (50–70 cm), horizontal viewing distance to the keyboard (63–82 cm), the height of the keyboard from the floor (60–82 cm), the light intensity between the participant and the computer (75–150 Cd/m^2^), and the light intensity of the room (200–500 Cd/m^2^), were recorded by using a digital light meter and small handheld meters (along with their expected values).

Next, the applicable viewing angles [viewing angle of the participant's eye level to the top of the computer screen (10°-20°), viewing angle of the participant's eye level to the center of the computer (21°-30°), and viewing angle of the participant's eye level to the bottom of the screen (31°-40°)] were calculated in degrees using the formula *Tanα* = *C*/*B*, where α equals the viewing angle, *B* is the horizontal viewing distance from the computer screen to the participant, and *C* is the viewing distance from the top of the computer screen to eye level. These recommended values were adapted from the study conducted in Ghana among university administrative staff (18).

### Operational definition

#### Computer vision syndrome

Computer vision syndrome (CVS) refers to a group of eye and vision-related problems that arise as a result of the prolonged use of devices with digital screens (39). Study participants had symptoms of eye burning; itching of the eye; feeling of a foreign body in the eye; eye tearing or watery eye; excessive eye blinking; eye redness; eye pain; heavy eyelids; eye dryness; blurred vision; double vision; the difficulty of focusing for near vision; increased sensitivities to light; colored halos around objects; feeling that sight is worsening; and headache for at least 1 week during the last 12 months. Participants who scored ≥six points using the formula of $\overset{16}{\underset{i=1}{\sum }}\begin{array}{c} \left(frequency\;of\;occurrence\right)\;\times \;\left(intensity\;of\;symptoms\right)\; \end{array}$ were considered to be positive for CVS; otherwise, they were considered to be negative for CVS. The presence of CVS was coded as “1,” whereas the absence of CVS was coded as “0” (1, 18, 38).

#### Good and poor workstation ergonomics

Any study participants who obtained ≥7 measurements in the recommended range out of 10 workstation ergonomic parameters assessed were considered to have good workstations; otherwise, they were considered to have poor workstations (18).

#### 20–20–20 ergonomics principle

After every 20 min on the device, look at a distant object at least 20 ft away for 20 s (40).

#### Blurred vision

“Indistinct, fuzzy visual images or a lack of sharpness of vision resulting in the inability to see fine detail” (19).

#### Taking regular health breaks

Take a brief break of 5 min after every hour between work (14).

#### Frequent eye blinking

It is a coping mechanism of blinking 12–18 times per minute (18).

#### Wearing non-prescription eyeglasses

Using non-correction eyeglasses while working behind a computer screen.

#### Using antiglare

Computer screens are covered with filters that prevent glare and reflections (20).

#### Adjusting computer brightness

Adjust computer brightness to the surrounding environment while using computers.

#### Adjustable sitting chair

Capable of adjusting the seat pan height so that the worker's legs are at right angles and feet rest flat on the floor (41).

#### Adjustable keyboard

Use the flattest and height-adjustable keyboard.

#### Glare at the workplace

The presence of glare or a high level of bright light due to direct light or reflections that disturb vision.

### Data quality assurance

To assure the quality of the data and its consistency, the questionnaires were prepared in English and translated into Amharic, the local working language, and again back-translated to English to ensure consistency. Before the actual data collection, the questionnaire was pretested among 24 (5%) CBE workers from non-selected districts and checked for clarity, understandability, and completeness. Finally, questionnaires that needed revision were revised by the principal investigator before being used for the final survey. For the workstation ergonomics assessment, a light meter was probed on working tables, and its reading was adjusted to record the distance between workers and their computer screen; study participants were instructed to sit down in their usual place.

The data collectors and supervisors were provided 2 days of training on the methods of data collection and the objective of the study before the data collection period. Four data collectors (2 BSc Optometry Nurses and 2 BSc Environmental and Occupational health professionals) and one supervisor (MPH in Epidemiology) were recruited. The Cronbach's alpha method (16 items = 0.87) was used to check the reliability and showed satisfactory internal consistency and high reliability of the questionnaire.

### Data processing and analysis

After data collection was completed, questionnaires were checked for completeness, coded, and entered into a database using EpiData software version 3.1. Then, they were exported, cleaned, and analyzed using SPSS version 26. For CVS outcomes, the severity score was computed by multiplying the frequency by the intensity of each 16 CVS symptoms, and then, it was given a score of 0, 1, 2, and 4. Later, a summation of the total severity score was calculated. Finally, the summation of the total severity score ≥6 was coded as “1” (Yes), and the total severity score of <6 was coded as “0” (No). Next, a descriptive statistical analysis (frequency and percentage) was utilized to describe the main dependent variable. Binary logistics regression was performed to determine variables associated with CVS. All assumptions of binary logistic regression were checked. Multicollinearity was checked using variance inflation factors (VIF); for all independent variables, VIF was <5, which indicates that there was an absence of multicollinearity. The Hosmer–Lemeshow test was performed to assess the model fitness and the *p*-value = 0.205, which indicates that the model was a good fit. Variables that had a *p*-value of < 0.2 during bivariable analysis were considered candidates for multivariable logistic regressions to control the potential confounders, and statistically significant variables were established at a *p*-value of < 0.05 in a multivariable logistics regression model. The strength and direction of association were measured and reported using an adjusted odd ratio (AOR) with a 95% confidence interval.

## Results

### Sociodemographic characteristics of study participants

A total of 458 bank workers participated with a response rate of 98.28%. Approximately 249 (54.4%) participants were male, and the majority of respondents, 193 (42.1%), were in the age groups of 31–40 years. Regarding marital status, 279 (60.7%) of the study participants were married. More than two-thirds (320, 69.9%) of the study participants were degree holders. A total of 301 (65.7%) of respondents had >5 years of work experience. The median gross incomes of respondents were ETB 15,570 (Table 1).

**Table 1:** Sociodemographic characteristics of workers of Commercial Bank of Ethiopia in Addis Ababa, Ethiopia, 2022 (n = 458).

**Variables**	**Category**	**Frequency**	**Percentage (%)**
Age (years)	20–30	172	37.6
	31–40	193	42.1
	≥41	93	20.3
Sex	Female	209	45.6
	Male	249	54.4
Work experience	<5 years	157	34.3
	≥5 years	301	65.7
Marital status	Single	142	31.0
	Married	278	60.7
	Divorced	35	7.6
	Widowed	3	0.7
Educational status	Certificate	3	0.7
	Diploma	3	0.7
	Degree	320	69.9
	Masters and above	132	28.8
Working position	Junior officer	150	32.8
	Senior officer	198	43.2
	Assistant manager	59	12.9
	Branch manager	51	11.1
Gross monthly income in ETB	<15,570	233	50.9
	≥15,570	225	49.1

### Personal and behavioral characteristics of study participants

More than three-fourths, i.e., 393 (85.8%), of study participants spent more than 7 h (>7) per day on computer screens. Nearly half, 233 (50.9%), of the study participants do not take regular health breaks, and nearly 356 (77.7%) of the study participants wear eyeglasses. Nearly half of the study participants, i.e., 256 (56.1%), took the task of frequent voluntary eye blinking. Approximately 283 (61.8%) study participants adjust computer brightness, and ~368 (80.3%) study participants do not use antiglare (Table 2).

**Table 2:** Personal and behavioral characteristics of workers of Commercial Bank of Ethiopia in Addis Ababa, Ethiopia, 2022 (n = 458).

**Variables**	**Category**	**Frequency**	**Percentage (%)**
Working hours per day on the computer screen	≤ 7 h	65	14.2
	>7 h	393	85.8
Number of working days per week on the computer screen	≤ 5 days	189	41.3
	>5 days	269	58.7
Have you heard about computer vision syndrome?	Yes	154	33.6
	No	304	66.4
Taking a regular healthy break	Yes	225	49.1
	No	233	50.9
Wearing non-prescription eyeglasses	Yes	102	22.3
	No	356	77.7
Taking frequent voluntary eye-blinking	Yes	256	56.1
	No	201	43.9
Having a history of previous eye illness	Yes	30	6.6
	No	428	93.4
Adjusting computer brightness to the surrounding environment	Yes	283	61.8
	No	175	38.2
Using antiglare for computer screen	Yes	90	19.7
	No	368	80.3
Using eye lubricant drops while working on a computer	Yes	29	6.3
	No	429	93.7
Frequency of taking a healthy break	Every 20 min	16	7.1
	Every 60 min	38	16.8
	Every 2 h	78	34.6
	≥2 h	93	41.3

### Ergonomics and environmental risk factors of study participants

More than half of the study participants, 272 (59.4%), reported the presence of glare, and ~291 (63.5%) study participants did not adhere to the 20–20–20 ergonomics principles. Assessment of workstation ergonomics during computer use revealed that 360 (78.6%) study participants had poor overall workstation ergonomics setup (Table 3).

**Table 3:** Ergonomics and environmental characteristics of workers of Commercial Bank of Ethiopia in Addis Ababa, Ethiopia, 2022 (n = 458).

**Variable**	**Category**	**Frequency**	**Percentage (%)**
Presence of glare in the working space	Yes	272	59.4
	No	186	40.6
Applying 20–20–20 ergonomics principle	Yes	167	36.7
	No	291	63.5
Overall workstation ergonomics setup	Good	98	21.4
	Poor	360	78.6
Using ergonomics adjustable sitting chairs	Yes	312	68.1
	No	146	31.9
Using adjustable keyboard	Yes	253	55.2
	No	205	44.8

### Workstation ergonomics assessment

Nearly half of the study participants, 242 (52.8%), had altered illumination from the recommended level. Nearly 283 (61.8%) respondents had compliance with the suggested distance from the center of the computer screen to the horizontal, and almost 95.6% of the study participants had recommended keyboard height from the floor. Approximately 55.7% of bank workers had deviated room light from the suggested value, and ~61.1% of bank workers maintain an eye to the center of the computer screen at a viewing angle that exceeds the recommended levels (Table 4).

**Table 4:** Measured workplace Ergonomics parameters among workers of Commercial Banks of Ethiopia in Addis Ababa, Ethiopia, 2022 (n = 458).

**Individual workstation ergonomics parameters**	**Category**	**Frequency**	**Percentage (%)**
Viewing angle from the top of the computer screen to the eye	Not altered	201	43.9
	Altered	257	56.1
Viewing angle from the center of the computer screen to the eye	Not altered	178	38.9
	Altered	280	61.1
Viewing angle from the bottom of a computer screen to the eye	Not altered	129	28.2
	Altered	329	71.8
Distance from the top of a computer screen to the horizontal	Not altered	139	30.3
	Altered	319	69.7
Distance from the bottom of a computer screen to the horizontal	Not altered	220	48.0
	Altered	238	52.0
Distance from the center of a computer screen to the horizontal	Not altered	283	61.8
	Altered	175	38.2
Viewing distance from keyboard to eye	Not altered	97	21.2
	Altered	361	78.8
Height keyboard from the floor	Not altered	439	95.6
	Altered	19	4.1
Room light	Not altered	222	44.3
	Altered	236	55.7
The intensity of light between the participant and the computer	Not altered	216	47.2
	Altered	242	52.8

### Prevalence of computer vision syndrome

**Figure 2:**
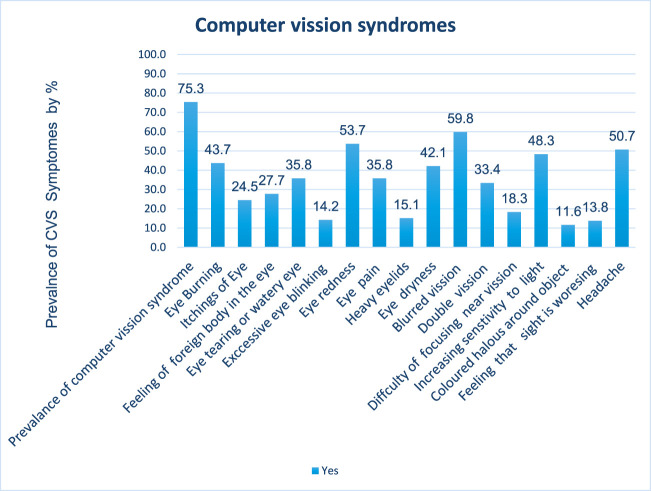
Percentage of overall and individual computer vision syndrome among workers of Commercial Banks of Ethiopia in Addis Ababa, Ethiopia, 2022.

The prevalence of self-reported CVS over 12 months was 75.3% (95% CI: 71.2–79.2) among bank workers of Commercial Bank Ethiopia. Most commonly reported CVS symptoms were blurred vision (59.8%), followed by eye redness (53.7%) and headache (50.7%), but excessive eye blinking (14.2%), feeling that sight is worsening (13.8%), and colored halos (11.6%) were irregularly reported (Figure 2).

### Factors associated with computer vision syndrome

After adjusting for potential confounders, four variables, the presence of glare in working space, 20–20–20 ergonomics principle, wearing non-prescription eyeglasses, and overall workstation ergonomic setup were independent predictors of CVS. The odds of having CVS among workers who had reported the presence of glare in the working space was 4.45 times greater than their counterparts (AOR = 4.45; 95% CI: 2.45–8.08). The study participants who did not follow 20–20–20 ergonomics interventions were two times more likely to develop CVS than workers who did follow 20–20–20 ergonomics interventions (AOR = 1.98, 95% CI: 1.06–3.68). The odds of CVS were four times higher among workers who were wearing non-prescription eyeglasses than their counterparts (AOR = 4.17; 95% CI: 1.92–9.06). Moreover, the bank workers who had poor workstation ergonomic setups were seven times more likely to have CVS than those who had good workstation ergonomic setup (AOR = 7.39; 95% CI: 4.06–13.49) (Table 5).

**Table 5:** Multivariable logistic regression analysis for computer vision syndrome and associated factors among computer user bank workers of CBE in Addis Ababa, Ethiopia, 2022 (n = 458).

**Variables**	**Category**	**CVS**		**COR (95% CI)**	**AOR (95% CI)**	***p*-value**
		**Yes**	**No**			
Working position	Junior officer	104	46	1	1	
	Senior officer	152	46	1.46 (0.90–2.35)	1.23 (0.439–3.49)	0.686
	Assistance	48	11	1.93 (0.92–4.05)	1.650 (0.42–6.45)	0.472
	Managers	41	10	1.81 (0.83–3.93)	1.16 (0.29–4.64)	0.830
Work experience (years)	<5 years	112	45	1	1	
	≥5 years	233	68	1.37 (0.88–2.13)	1.31 (0.46–3.75)	0.605
Weekly computer usage during the day	≤ 5 days	129	60	1	1	
	>5 days	216	53	1.89 (1.23–2.91)	1.38 (0.79–2.40)	0.255
The presence of glare in the working space	Yes	242	30	6.5 (4.03–10.47)	4.45 (2.45–8.08)	0.0001^***^
	No	103	83	1	1	
Taking a regular healthy break	Yes	147	78	1	1	
	No	198	35	3.00 (1.91–4.71)	1.43 (0.77–2.65)	0.250
Follow the 20–20–20 ergonomic principle	Yes	99	68	1	1	
	No	246	45	3.75 (2.41–5.84)	1.97 (1.06–3.67)	0.031^*^
Wearing non-prescription eyeglasses	Yes	87	15	2.20 (1.21–3.99)	4.17 (1.92–9.06)	0.0001^***^
	No	258	98	1	1	
The habit of voluntary eye blinking	Yes	152	75	1	1	
	No	193	38	2.50 (1.607–3.908)	1.15 (0.63–2.09)	0.643
Adjusting computer brightness	Yes	194	89	1	1	
	No	151	24	2.88 (1.753–4.75)	1.48 (0.77–2.82)	0.233
Using antiglare or blue filter	Yes	51	39	1	1	
	No	294	74	3.03 (1.86–4.95)	1.82 (0.93–3.54)	0.076
Overall workstation ergonomics setup	Good	40	58	1		
	Poor	305	55	8.04 (4.90–13.18)	7.39 (4.05–13.49)	0.0001^***^

1 = reference.

^*^Significant at a *p*-value of < 0.05.

^***^Significant at a *p*-value of < 0.01.

## Discussion

This study revealed that a 12-month self-reported overall prevalence of CVS among workers of the Commercial Bank of Ethiopia was 75.3% (95% CI = 71.2, 79.2). Since the majority of prior studies in Ethiopia defined CVS as having at least one symptom, it is challenging to directly compare this result with those of other studies. However, this finding was nearly consistent with other studies conducted in different countries, 74.3% in Spain (38); 74% in Abuja, Nigeria (42); 73% in Gondar, Ethiopia (22); 73.9% at Gondar University, Ethiopia, (24); 75.6% in Jimma University, Ethiopia (23); and 74.6% in Addis Ababa, Ethiopia, (11). On the other hand, our findings were higher than studies conducted in Nigeria (29.3%) (43); southern Malaysia (68.1%) (30); Sri Lankan, (67.4%) (4); Ghana (51.5%) (18); Debre Tabor, Ethiopia (69.5%) (1); Addis Ababa, Ethiopia (68.8%) (20); and Ethiopian University instructors (70.4%) (25). The possible reason might be due to the differences in sample size and study participants. Study participants in the aforementioned studies were university administrative and academic staff who might be aware of about health consequences of continuous computer use, which could lower the prevalence of CVS. Besides, office workers and secretary workers might have less workload and contact hours with the computer than a banker or secretary workers and working at a minister's office with ideal working environments.

In contrast, our study indicated a reduced prevalence of CVS compared to other studies conducted in Malaysia (89.9%) (15); Karnataka, India (83.5%) (44); Tamil Nadu, India (81.9%) (14); and Addis Ababa Ethiopia (81.3%) (21). The possible explanation could be that medical and engineering university students could partly play a role in experiencing CVS because students may be under more pressure to complete their coursework and projects (11). On the other hand, bank workers might have regular work schedule periods to give service to customers. Regarding a study done in Ethiopia, the observed higher prevalence of CVS could be due to the inclusion of postural or non-ocular symptoms in the definitions of CVS (21). However, in this study, only visual and ocular symptoms, including headache, were considered. In addition, a duration of symptom specification that lasted 1 week was considered to define CVS, but in some of the aforementioned studies, no duration of symptom specification was considered. These disparities could account for the reported higher prevalence of CVS in Malaysia, in Karnataka and Tamil Nadu, India, and in Addis Ababa, Ethiopia than in our study (14, 15, 21, 44).

Our study findings revealed that study participants who reported the presence of glare at the workplace were significantly associated with increased magnitudes of CVS than their counterparts. This result is in agreement with other studies conducted in Ghana among undergraduate students (6). These findings might be due to the fact that employees who report the presence of glare at their workstations may experience eye discomfort and stress more frequently than their counterparts, and bank offices are frequently situated along busy roadsides for customer convenience, which could result in unnecessary reflection from parking and passing vehicles. Besides the presence of glare at their workstation, wearing non-prescribed eyeglasses while working at computer screens was significantly associated with higher CVS prevalence. This result is in line with other studies in Saudi Arabia and Ethiopia (22, 45). These findings could be explained by the fact that bankers who use occupational eyeglasses that are non-professionally recommended have no radiation protection capacity or that workers who use none correction eyeglasses must bend forward to look at computer screens, which will make it harder to focus on monitor screens (22).

The 20–20–20 ergonomic principle was identified as an important predictor for CVS. The odds of CVS were two times higher among bank workers who did not adhere to the 20–20–20 ergonomic principle than their counterparts. This result is consistent with other studies conducted in different countries, in Medan, Indonesia, and Jeddah, Saudi Arabia (34, 40). The possible explanation could be that computer screens are made up of pixels that start to brighten at the center and dim outward, making it impossible for the human eye to maintain focus (46). In contrast, practicing the 20–20–20 rule, which requires people to look at distant objects for 20 s every 20 min, may encourage people to frequently blink their eyes, stretch their arms, and even drink water. As a result, bank employees will stay hydrated and experience less eye irritation as well as relief from CVS symptoms.

Similarly, poor overall workstation ergonomics office setup was significantly associated with increased CVS than those who had good workstation ergonomics. This finding was supported by studies done in developing countries and Ghana (4, 18), which indicates that poor workstation ergonomics could result in a lack of compliance with recommended visual ergonomic practices, and this departure might lead computer users to develop CVS. In addition, nearly 78.6% of the study participants were found to be working in poor office workstation ergonomics design, which is consistent with prior research findings from Ghana (18). The reason behind this claim could be that workstation ergonomic arrangements comparable to those already in place could be anticipated. However, in Sri Lanka, in a study of workstation evaluation, 88.4% were non-compliant with the recommended value (4). This surpasses our findings; tool differences used in the assessment of workstation ergonomics could be one possible explanation.

## Strengths and limitations of the study

Self-reported questionnaires were utilized to measure CVS. To overcome recall bias, we used a standardized tool (CVS-Q) to reduce the unintended consequences of self-reported measurement; and onsite workstation ergonomics assessments were conducted for data collection. However, the blue light radiation that computers emit was not monitored at display units, and similar times of day were not taken into account during workstation light assessment. Future researchers have to address this issue, and a comparative cross-sectional study shall be conducted.

## Conclusion

We found that three in four bank workers in Ethiopia developed CVS, which calls for urgent intervention. Poor workstation ergonomics setups, the presence of glare, not following the 20–20–20 principle, and wearing any noncorrection eyeglasses were identified as the determinant factors of CVS. Therefore, the government, bank managers, and other concerned bodies should implement a comprehensive intervention strategy, including creating awareness about poor workstation ergonomic setup and the negative health effects of glare on the eye. The bank workers should also adhere to the 20–20–20 ergonomic principle.

## Data availability statement

The raw data supporting the conclusions of this article will be made available by the authors, without undue reservation.

## Ethics statement

Ethical approval of the research was obtained from Addis Ababa University, Research Ethics Committee of the School of public health (SPH) with Ref. No. SPH/1321/14. After explaining the purpose of the study, each participant provided verbal informed consent for their willingness and cooperation to provide the necessary information, voluntary participation was appreciated.

## Author contributions

KG: Conceptualization, Data curation, Formal analysis, Investigation, Methodology, Project administration, Software, Validation, Visualization, Writing—original draft, Writing—review & editing. AB: Formal analysis, Validation, Writing—review & editing. TA: Formal analysis, Software, Supervision, Writing—review & editing. YZ: Software, Supervision, Validation, Writing—review & editing. CD: Conceptualization, Data curation, Formal analysis, Methodology, Software, Supervision, Validation, Writing—review & editing.
