# Supplementary material for: Computer vision syndrome and ergonomic risk factors among workers of the Commercial Bank of Ethiopia in Addis Ababa, Ethiopia: an institutional-based cross-sectional study
Source: Front Public Health. 2024 May 9;12:1341031. doi: 10.3389/fpubh.2024.1341031 (PMC11111856; doi:10.3389/fpubh.2024.1341031)
Supplement: Supplementary file 1 [file Data_Sheet_1.docx]

**Computer Vision Syndrome and Ergonomic Risk Factors Among Workers of the Commercial Bank of Ethiopia in Addis Ababa, Ethiopia: An Institutional-Based Cross-Sectional Study**

Kassahun Ayele Gasheya^1*^, Azanaw Asega Belay^1^, Teferi Abegaze^2^ and Yifokire Tefera Zele^2^ and Chala Daba^3^

^1^Department of Occupational Health and Safety, College of Medicine and Health Science, Wollo University, Dessie, Ethiopia.

^2^Department of Preventive Medicine, School of Public Health, Addis Ababa University, Addis Ababa, Ethiopia.

^3^Department of Environmental Health, College of Medicine and Health Science, Wollo University, Dessie, Ethiopia

# * Correspondence: [astedekassahun@gmail.com](mailto:astedekassahun@gmail.com)

**Part I- Socio-Demographic Information related questionaries**

1. Age:____________ (years)
2. Sex: 1) Male 2) Female
3. How many years did you working in banks (years) ________
4. What is your marital status?
5. Single 2. Married 3. Divorced 4. Widowed 5. Others
6. Educational status: 1. Certificate 2. Diploma 3. Degree 4. Masters and above
7. Monthly income /salary in ETB Birr___________________
8. What is your current working position?
   1. junior customer business officer
   2. Senior customer business officer
   3. Assistance branch manager
   4. Branch manager

**Part II-Personal and behavioral factors related questionaries**

8. How many hours per day do you work on computer devices (hours/day? ________

9. How many days per week do you use computer devices (day/week? ____________

10. Duration of computer use (years)? ______________

11. Is there any glare at your workplace (“presence of high levels bright light that disturbs the vision due to direct or reflected sunlight or overhead lamps”) on your computer devices screen? 1. No 2. Yes

12. Have you heard about computer vision syndrome? 1. No 2. Yes

13. Did you take a brief healthy break of five minutes for every hour between works while using computer devices? 1. No 2. Yes

14. If “Yes Q13”, how often do you take a break while using computer devices?

1. Every 20 minutes of work
2. Every 60 minutes of work
3. Every 2 hours of work
4. More than every 2 hours

15. How long do you take a healthy break?

1. 1- 10 minutes 2. 11-20 minutes 3. >=21 minute

16. If “No Q13”, do you follow 20- 20- 20 ergonomics principles (Every 20 minutes on the device look 20 feet away for 20 seconds) 1. No 2. Yes

17. Do you use non-prescription eyeglasses while working on computer devices? 1. No 2. Yes

18. If “yes Q17, does your eyeglasses contain anti-reflecting or blue light filter coating? 1. No 2. Yes

19. If ‘No Q17”, do you use contact lenses while using a computer device? 1. No 2. Yes

20. Do you have a habit of frequent eye blinking (12-18 times eye blinking per minute)?

1. No 2. Yes

21 Do you adjust the contrast of your computer devices with the surrounding brightness?

1. No 2. Yes

22. Do you use an anti-glare / screen filter / blue light filter for your computer screen?

1. No 2. Yes

23. Do you use lubricant eye drops while working on the computer devices?

1. No 2. Yes

24. If “Yes Q23”, how frequently do you use lubricant eye drops while working on the computer devices? 1. Always 2. Often 3. Sometimes 4. Rarely 5. Never

25. If “No Q23”, do you use an adjustable keyboard? 1. No 2. Yes

26. Do you use an adjustable chair? 1. No 2. Yes

27. Do you have a previous history of eye illness? 1. No 2. Yes

**Part III- workstation Ergonomics Parameters measurements**

1. The viewing distance from the horizontal to the top of the screen_________________ in Centimeter (Cm) verse (18–28 cm).
2. The viewing distance from the horizontal to the bottom of the screen ___________________ in Centimeter (Cm) verse (40–60 cm).
3. The viewing distance from the eye to the horizontal center of the screen___________in Centimeter (Cm) verse (50–70 cm).
4. Viewing distance from the eye to the keyboard ______________ in Centimeter (Cm) verse (63–82 cm).
5. Height of the keyboard from the floor _____________in Centimeter (Cm) verse (60–82 cm).
6. The viewing angle of the participant’s eye level to the top of the computer screen _____________in degree verse (10°–20°).
7. The viewing angle of the participant’s eye level to the center of the computer screen _____________ in degree verse (21°–30°).
8. The viewing angle of the participant’s eye level to the position of the bottom of the screen_________________ in degree verse (31°–40°).
9. Light intensity between participant and computer at left(L) ________ middle (M)_______right (R) _______Average__________ in Lux verse (75–150 Cd/m2).
10. The light intensity of room__________ in Lux verse (200–500 Cd/m2).

**Part IV- Computer Vision Syndrome Related Questionaries**

1. First, the **frequency**, that is, how often the symptom occurs, considering that:

**Never** = the symptom does not occur at all

**Occasionally** = sporadic episodes or once a week

**Often or always**= 2 or 3 times a week or almost every day.

1. Second, the **intensity** of the symptom: if you indicated **never for frequency, you should not mark anything for intensity**.

| **Computer vision syndrome symptoms** | **Frequency** | | | **Severity/intensity** | |
| --- | --- | --- | --- | --- | --- |
|  | Never  (0) | Occasionally  (1) | Often or Always (2) | Moderate  (1) | Intense  (2) |
| 1 Burning eye | 0 | 1 | 2 | 1 | 2 |
| 2 Itching eye | 0 | 1 | 2 | 1 | 2 |
| 3 Feeling of a foreign body in eye | 0 | 1 | 2 | 1 | 2 |
| 4 Tearing | 0 | 1 | 2 | 1 | 2 |
| 5 Excessive blinking | 0 | 1 | 2 | 1 | 2 |
| 6 Eye redness | 0 | 1 | 2 | 1 | 2 |
| 7 Eye pain | 0 | 1 | 2 | 1 | 2 |
| 8 Heavy eyelids | 0 | 1 | 2 | 1 | 2 |
| 9 Dryness | 0 | 1 | 2 | 1 | 2 |
| 10 Blurred visions | 0 | 1 | 2 | 1 | 2 |
| 11 Double vision | 0 | 1 | 2 | 1 | 2 |
| 12 Difficulty of focusing for near vision | 0 | 1 | 2 | 1 | 2 |
| 13 Increased sensitivities to light | 0 | 1 | 2 | 1 | 2 |
| 14 Colored halos around objects | 0 | 1 | 2 | 1 | 2 |
| 15 Feeling that sight is worsening | 0 | 1 | 2 | 1 | 2 |
| 16 Headache | 0 | 1 | 2 | 1 | 2 |

**Score ∑(frequency of symptom occurrence)_i_ x (intensity of symptom)_i_, if the total score of 16 questions is ≥6 points, the bank worker is considered as having computer vision syndrome.**
